# Supplementary material for: Characterization and Transcriptional Regulation of the 2-Ketogluconate Utilization Operon in Pseudomonas plecoglossicida
Source: Microorganisms. 2024 Dec 8;12(12):2530. doi: 10.3390/microorganisms12122530 (PMC11678583; doi:10.3390/microorganisms12122530)
Supplement: Supplementary file 1 [file microorganisms-12-02530-s001.zip › microorganisms-3307364-supplementary.pdf]

Characterization and transcriptional regulation of the 2-ketogluconate utilization operon in *Pseudomonas plecoglossicida*

Supplementary materials

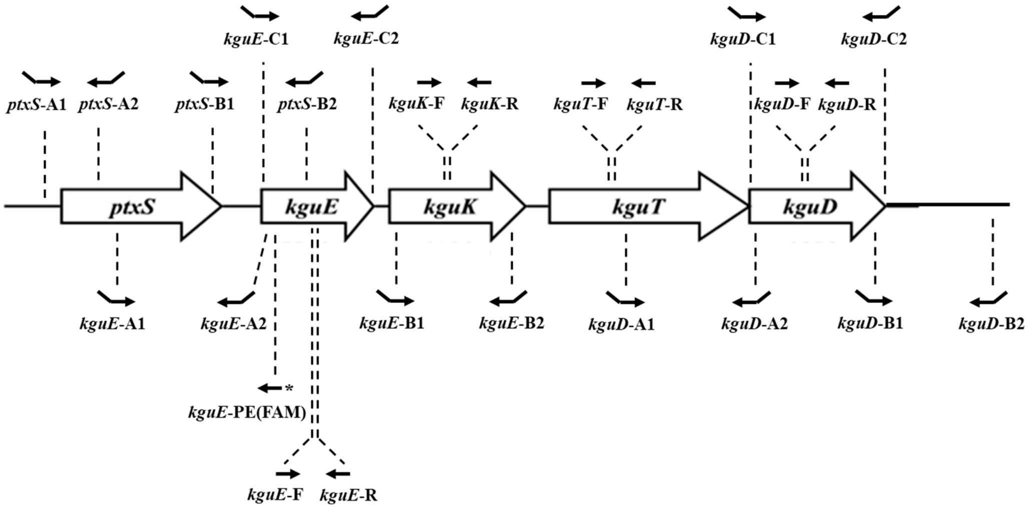

Figure S1. Graphical representation of some primers used in this study

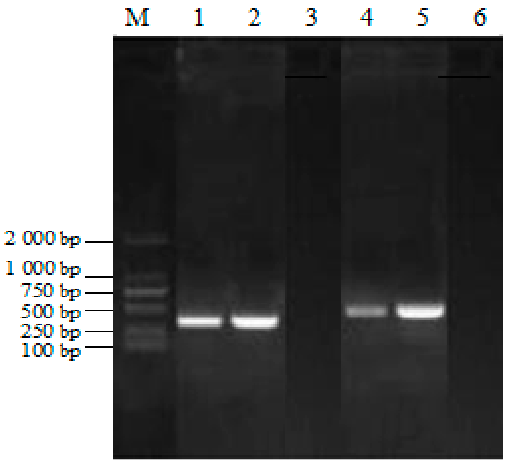

Figure S2. Electrophoretic analysis of the *kguE*-*kguK* and *kguK*-*kguT* transgene regions amplification with different templates

M: DL 2,000 DNA Marker; Lane 1: the *kguE*-*kguK* transgene region amplification with cDNA as

template; Lane 2: the *kguE*-*kguK* transgene region amplification with genome DNA as template (positive control); Lane 3: the *kguE*-*kguK* transgene region amplification with ddH_2O as template (negative control); Lane 4: the *kguK*-*kguT* transgene region amplification with cDNA as template; Lane 5: the *kguK*-*kguT* transgene region amplification with genome DNA as template (positive control); Lane 6: the *kguK*-*kguT* transgene region amplification with ddH_2O as template (negative control)

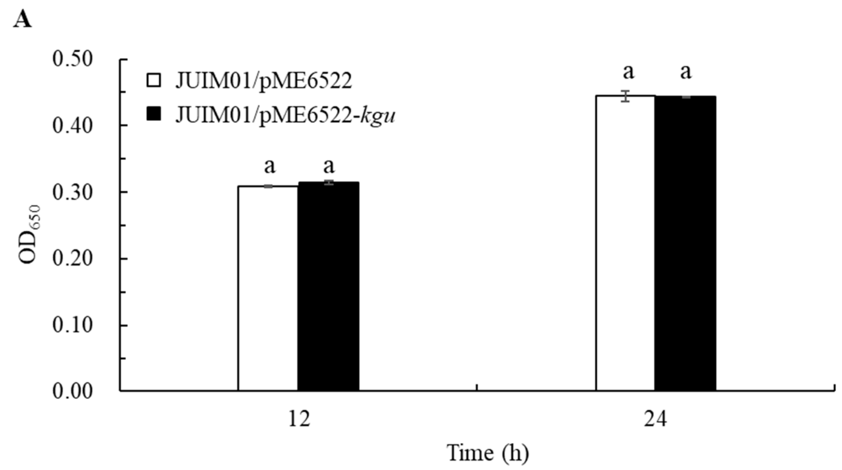

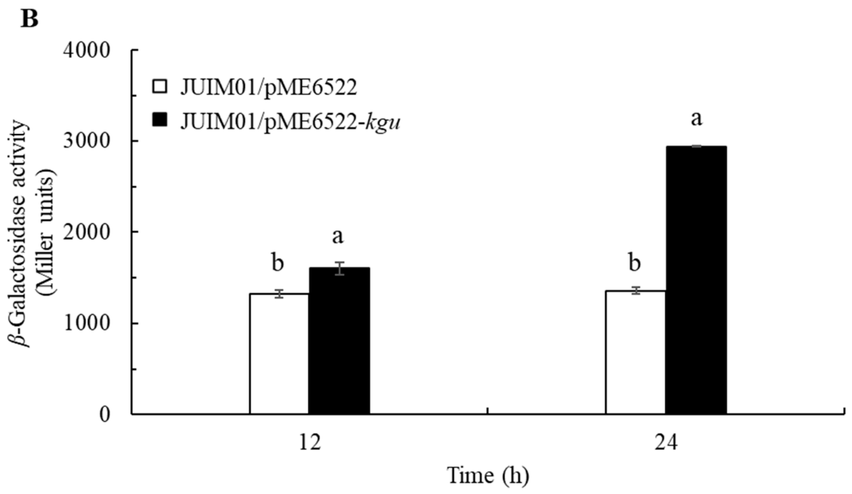

Figure S3. Comparison of (A) the growth and (B) the *β*-galactosidase activities between the recombinants JUIM01/pME6522 and JUIM01/pME6522-*kgu* after 12-/24-h cultivation

The different superscript letters indicate significant differences (*P*<0.01) between the corresponding values

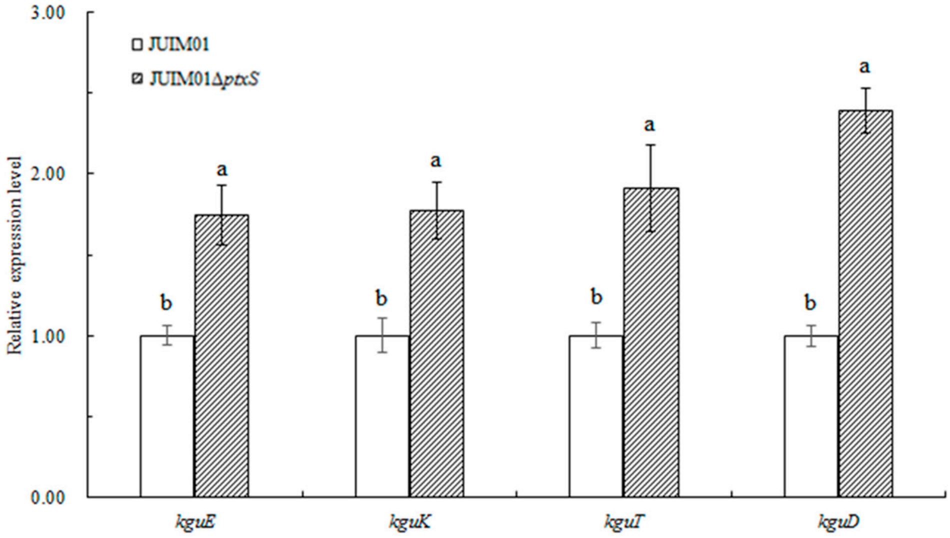

Figure S4. Comparison of the relative expression levels of the *kgu* operon in *P. plecoglossicida* JUIM01 and JUIM01Δ*ptxS*

The different superscript letters indicate significant differences (*P*<0.01) between the corresponding values

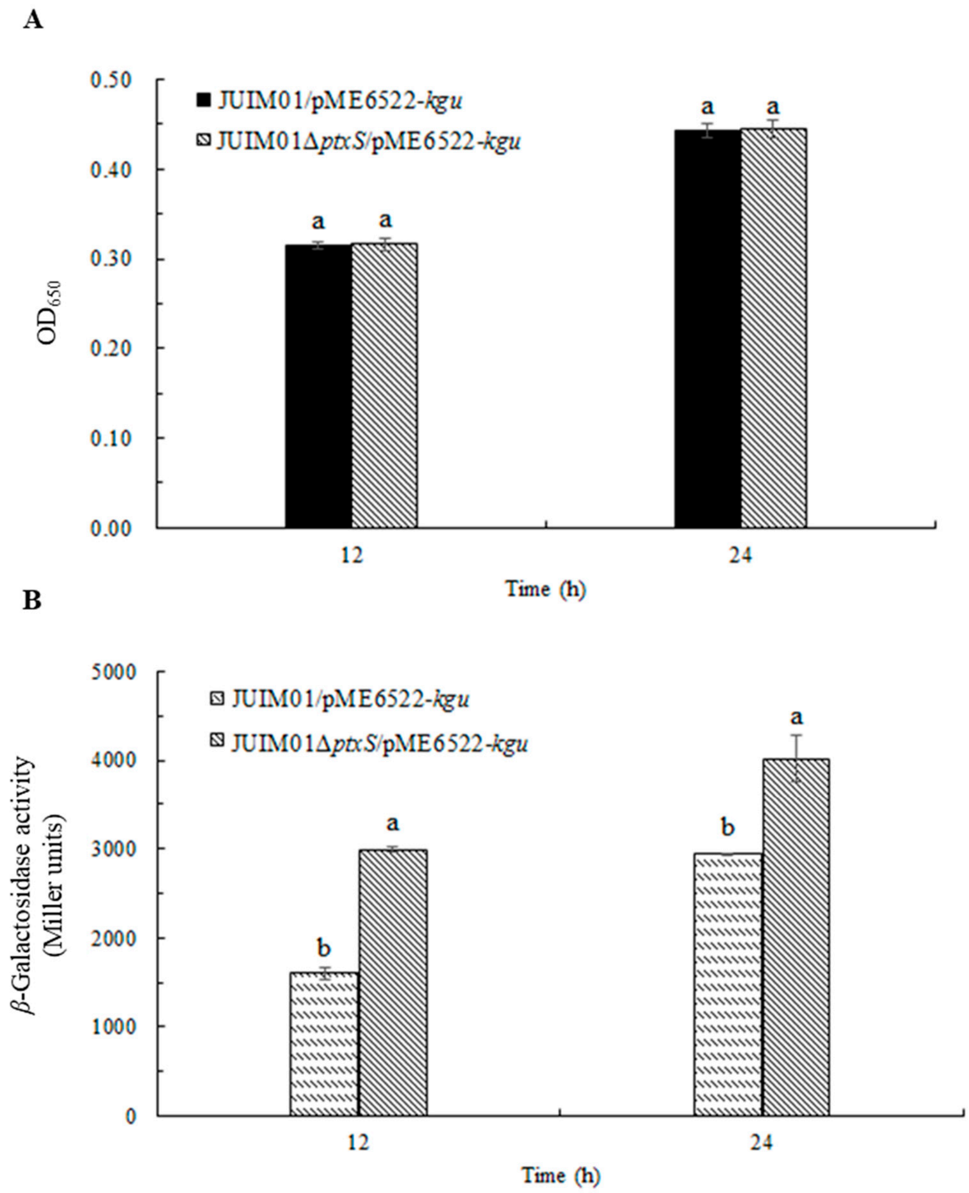

Figure S5. Comparison of (A) the growth and (B) the *β*-galactosidase activities between the recombinants JUIM01/pME6522-*kgu* and JUIM01Δ*ptxS*/pME6522-*kgu* after 12-/24-h cultivation The different superscript letters indicate significant differences (P<0.01) between the corresponding values

Table S1 The primers for construction of the knockout and complemented strains

Primers	Sequence (5'-3')	Description (Restriction sites)
kguE-A1	CGGGATCCAGCACTTGCAGGCGTTGCAG	To amplify the upstream homologous fragment of the *kguE* gene (*Bam*H I) for *kguE* knockout
kguE-A2	TGGACCTGCTGGCGGGTGTAGCGAAGCTCGACAGGCTGATGG	
kguE-B1	CCATCAGCCTGTCGAGCTTCGCTACACCCGCCAGCAGGTCCA	To amplify the downstream homologous fragment of the *kguE* gene (*Hin*d III) for *kguE* knockout
kguE-B2	CCCAAGCTTTTCGGTACCGCGCGCATCACG	
kguD-A1	CCGGAATTCCAGCTGGCTGAAGGCAC	To amplify the upstream homologous fragment of the *kguD* gene (*Eco*R I) for *kguD* knockout
kguD-A2	GTTATCCACCGCACAACGCTCGACCAGGGTCACCTC	
kguD-B1	GAGGTGACCCTGGTCGAGCGTTGTGCGGTGGATAAC	To amplify the downstream homologous fragment of the *kguD* gene (*Bam*H I) for *kguD* knockout
kguD-B2	CGCGGATCCGGTATTGAGCCAGAGCATG	
ptxS-A1	ATGGAATTCGCATCTACGGCGACCTCAGC	To amplify the upstream homologous fragment of the *ptxS* gene (*Eco*R I) for *ptxS* knockout
ptxS-A2	TGAGCCAAGGCAGTGATGCCGAGCTGCTCGATCA	
ptxS-B1	TGATCGAGCAGCTCGGCATCACTGCCTTGGCTCA	To amplify the downstream homologous fragment of the *ptxS* gene (*Hin*d III) for *ptxS* knockout
ptxS-B2	ATTAAGCTTCAGGTCGCAGGTGTCGCGGT	
kguE-C1	CCGCTCGAGATAAAGGAGGCCGACCATGCATGCGAACCCTGT	To amplify the insert containing *kguE* gene and restriction sites (*Xho* I and *Bam*H I) for *kguE* complementation
kguE-C2	CGCGGATCCTCAGCCATGGTGGACCT	
kguD-C1	CCCAAGCTTATGAAAAAACGCATCGTCCTG	To amplify the insert containing *kguD* gene and restriction sites (*Hind* III and *Eco*R I) for *kguD* complementation
kguD-C2	CCGGAATTCTTAACTGGCCCTGCGCTCCC	

Table S2 Main primers used in this study

Primers	Sequence (5'-3')	Description
P1	ATGGTCCACTTCATGCCCG	To amplify the *kguE*-*kguK* intergenic region
P2	CCAGCGAATCGTCTCCCAC	
P3	AGGTGGTGGACACGGTAGG	To amplify the *kguK*-*kguT* intergenic region
P4	TGATATGCAGGTCATCGGCCA	
K1	ATGGCAAAAGCTTCGAATTCGCT GCAAGGCAGTCAATTGC	To amplify the *ptxS*-*kguE* intergenic region with 20-bp homologous arms to pME6522 vector on both sides
K2	ATCCGCTCACAATTCTGCAGTTT CATTTCCGCGCAAAAAACAG	
kguE-PE(FAM)	AACAGCGGCAGGTAGGCGACC	A specific probe to the *kgu* operon promoter region for primer extension. (FAM) represents the probe is labeled with FAM at its 5' end
kguE-F	GCTGTTCGTGGAGAACGAC	Primers for RT-qPCR of *kguE* gene
kguE-R	CGATGTCGAAGGTCATGC	
kguK-F	GCGACCCGCAAGTGGAATAC	Primers for RT-qPCR of *kguK* gene
kguK-R	GAAGGAGATGCTGCGACCGT	
kguT-F	GATCTACCTCTGCCACTGGTTC	Primers for RT-qPCR of *kguT* gene
kguT-R	TTGACCAAGTAGCCGGACA	
kguD-F	CCGAAACCACTGCCGACACC	Primers for RT-qPCR of *kguD* gene
kguD-R	ACGATGCCCAGCGTCTTGC	
rpoD-F	GATTCGTCAGGCGATCAC	Primers for RT-qPCR of the housekeeping gene *rpoD*
rpoD-R	AATACGGTTGAGTTGTTGA	
